# Regulatory networks of post-translational modifications in diabetic kidney disease: from pathogenic mechanisms to therapeutic frontiers

**DOI:** 10.3389/fendo.2026.1818156

**Published:** 2026-06-23

**Authors:** Qing Jiang, Deqi Meng, Shenglong Zhu, Wei Tang, Siyuan Cui

**Affiliations:** 1 Department of Endocrinology, Nanjing Medical University, The Affiliated Wuxi People’s Hospital of Nanjing Medical University, Wuxi, China; 2 Department of Endocrinology, The Affiliated Geriatric Hospital of Nanjing Medical University, Nanjing, China; 3 Wuxi School of Medicine, Jiangnan University, Wuxi, China

**Keywords:** diabetic kidney disease, epigenetics, post-translational modifications, renal fibrosis, targeted therapy

## Abstract

Diabetic kidney disease (DKD) remains a principal microvascular complication of diabetes, driven by a multifaceted pathogenesis that extends beyond pure metabolic dysregulation. Post-translational modifications (PTMs) have emerged as pivotal mechanisms that dynamically regulate protein function and stability under diabetic stress. This review provides a comprehensive synthesis of the regulatory networks governing PTMs in DKD, systematically dissecting the molecular landscapes of phosphorylation, acetylation, ubiquitination, glycosylation, methylation, and SUMOylation. Beyond clarifying individual pathogenic mechanisms, the dysregulation of PTM networks links systemic metabolic disturbances to persistent renal injury. Furthermore, we evaluate emerging therapeutic strategies designed to modulate these modifications. We conclude that targeting PTMs represents a critical advance in DKD therapy, shifting from broad symptomatic management to precise molecular intervention—offering new directions for arresting the progression of DKD.

## 1 Introduction

Diabetic Kidney Disease (DKD), a detrimental microvascular complication of diabetes mellitus (DM), affects 30-40% of diabetic patients globally (1). Its insidious progression begins with microalbuminuria and, without intervention, advances to end-stage renal disease (ESRD), the leading cause of renal failure worldwide (2). DKD pathogenesis is complex, involving genetic and environmental factors, metabolic disorders, hemodynamic alterations, oxidative stress, inflammation, and renal fibrosis (3, 4). Current strategies have failed to effectively halt DKD progression, highlighting the urgent need for novel molecular targets.

Post-translational modifications (PTMs) are fundamental, dynamic, and often reversible covalent modifications of protein amino acid residues, occurring enzymatically or non-enzymatically after translation to regulate protein function (5). PTMs vastly expand proteome functional diversity and include classic modifications (phosphorylation, acetylation, ubiquitination, glycosylation, methylation) and emerging types (succinylation, lactylation) (6). By altering protein conformation, activity, localization, and interaction networks, PTMs systemically influence nearly all cellular processes, including metabolic reprogramming and gene expression (7).

**Figure 1:**
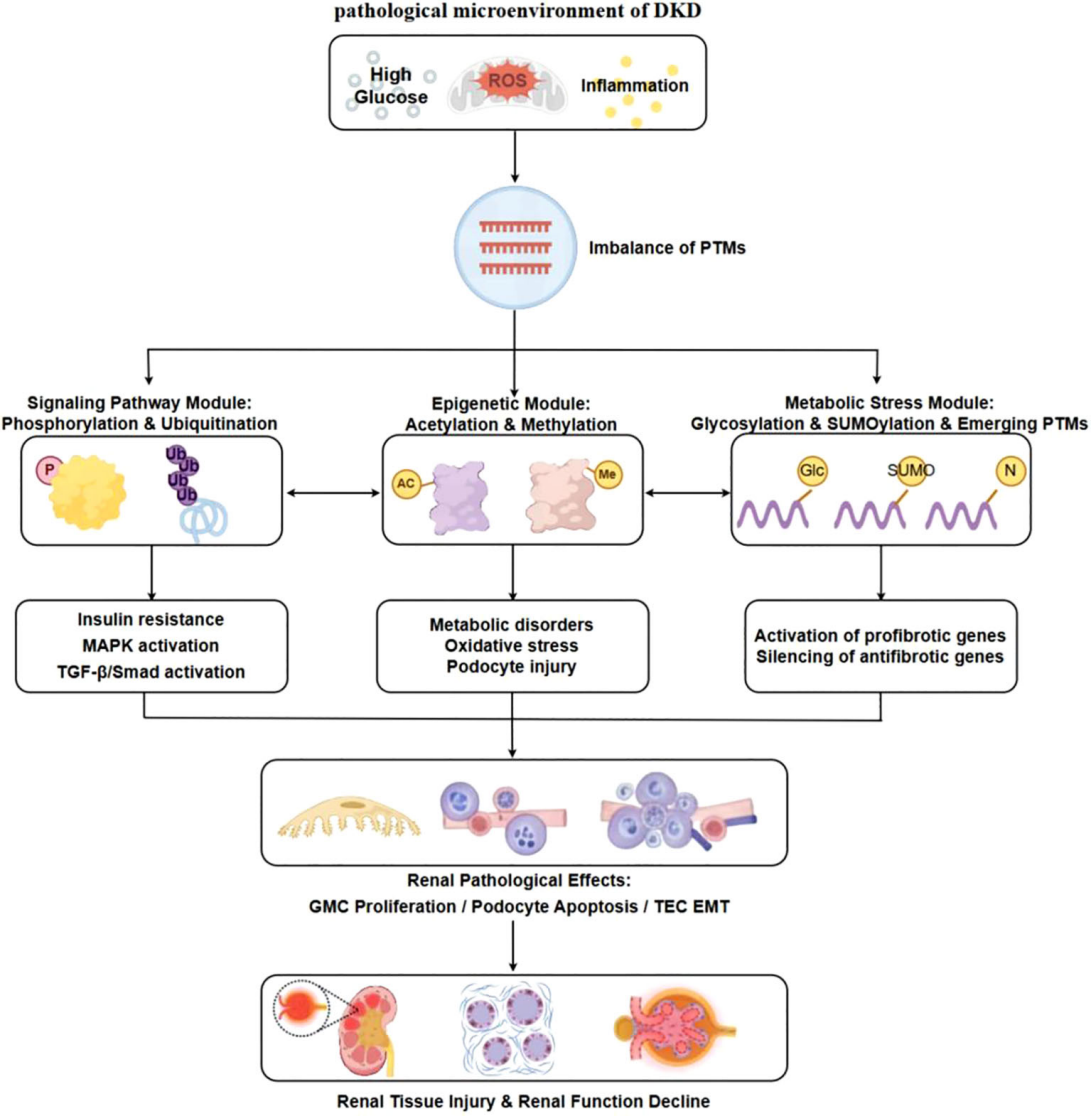
Schematic representation of PTM−mediated pathogenesis in DKD. Hyperglycemia and metabolic disturbances cause systemic dysregulation of PTM networks. Signaling module (phosphorylation/ubiquitination) induces insulin resistance and activates MAPK/TGF−β/Smad pathways. Epigenetic module (acetylation/methylation) remodels chromatin and promotes oxidative stress and podocyte injury. Metabolic module (glycosylation/SUMOylation/emerging PTMs) regulates fibrotic gene expression. Together, these processes lead to GMC proliferation, podocyte apoptosis, TEC EMT, renal injury, and progressive dysfunction. Created with Figdraw.

Recent research reveals that dynamic PTM network dysregulation is central to DKD progression (8, 9). Pathogenic microenvironments—such as high glucose (HG), advanced glycation end products (AGEs), and oxidative stress—cause significant PTM profile alterations in intrinsic renal cells. These include glomerular mesangial cells (GMCs), podocytes, and tubular epithelial cells (TECs). These changes are active pathological drivers that modulate key pathways (e.g., Wnt/β-catenin, TGF-β/Smad) to drive ECM (extracellular matrix) imbalance, aberrant inflammation, and cellular phenotypic transitions, promoting DKD (10, 11). Certain PTMs, particularly epigenetic ones, may form the molecular basis of “metabolic memory”, encoding transient hyperglycemic exposure into a persistent pathological state. Therefore, investigating DKD’s PTM regulatory networks is essential for understanding disease mechanisms and developing precise therapeutic strategies targeting PTM-modifying enzymes (**Figure 1**).

## 2 Key PTM regulatory networks in DKD pathogenesis

### 2.1 Phosphorylation

Phosphorylation, a ubiquitous PTM mediated by the opposing actions of protein kinases and phosphatases, precisely regulates protein activity. While phosphorylation networks are finely tuned in healthy kidneys, their dysregulation across multiple signaling pathways is a core molecular event driving DKD progression.

#### 2.1.1 Aberrant phosphorylation in the insulin signaling pathway

The insulin signaling cascade is central to glucose homeostasis and renal integrity (12). Under physiological conditions, insulin binding to the insulin receptor (IR) triggers phosphorylation of tyrosine (Tyr) residues within the intracellular domain of IR, which subsequently recruits and activates the insulin receptor substrate (IRS) family proteins. Under diabetic conditions, the high glucose environment, through mechanisms such as oxidative stress, leads to a marked reduction in tyrosine phosphorylation levels of IR and IRS, accompanied by a compensatory increase in phosphorylation at serine (Ser)/threonine (Thr) sites, ultimately resulting in insulin resistance (13). This aberrant pattern, seen in DKD patients and models, inhibits the PI3K/AKT pathway, diminishing glucose uptake in podocytes and tubular cells and exacerbating apoptosis and inflammation (14).

#### 2.1.2 Hyperactivation of the MAPK pathway via phosphorylation cascades

The Mitogen-activated protein kinase (MAPK) superfamily mediates cellular stress, inflammation, proliferation, and apoptosis (15). In DKD, stimuli (AGEs, ROS, cytokines)40 activate ERK1/2, JNK, and p38 MAPK via characteristic dual-site phosphorylation (Thr202/Tyr204, Thr183/Tyr185, and Thr180/Tyr182, respectively) (16, 17). Significant crosstalk exists; for instance, activated JNK can directly phosphorylate inhibitory Ser residues on IRS-1, linking oxidative stress to insulin resistance in a vicious cycle. Activated MAPKs then phosphorylate downstream transcription factors, upregulating inflammatory mediators, cytokines, and ECM components, thereby driving renal injury and fibrosis (18).The mitogen-activated protein kinase (MAPK) pathway includes three major cascades: the extracellular signal-regulated kinase (ERK), c-Jun N-terminal kinase (JNK), and p38 MAPK pathways, which play critical roles in cellular stress responses, inflammation, proliferation, and apoptosis. In the context of DKD, HG-induced accumulation of AGEs, reactive oxygen species (ROS), and pro-inflammatory cytokines triggers a cascade reaction that leads to dual-site phosphorylation and activation of ERK1/2 (Thr202/Tyr204), JNK (Thr183/Tyr185), and p38 MAPK (Thr180/Tyr182) (19). Activated MAPKs subsequently phosphorylate downstream transcription factors such as activator protein-1 and nuclear factor kappa-B (NF-κB) (20, 21), thereby regulating the expression of related genes, promoting the synthesis and secretion of inflammatory mediators, cytokines, and ECM, ultimately leading to renal injury and fibrosis.

#### 2.1.3 Dysregulated phosphorylation in the TGF-β/Smad signaling pathway

The TGF-β/Smad pathway, a core driver of renal fibrosis, is critically dependent on phosphorylation. The HG environment promotes TGF-β overactivation, enhancing C-terminal Ser phosphorylation of Smad2 and Smad3 (22). Phosphorylated Smad2/3 complex with Smad, translocate to the nucleus, and bind to Smad-binding elements, potently activating pro-fibrotic gene transcription. This accelerates ECM deposition in glomeruli and the tubulointerstitium (23). Non-coding RNAs (miRNAs, lncRNAs) also contribute by indirectly modulating the phosphorylation status of pathway members (24, 25).

### 2.2 Acetylation

Acetylation is an enzymatic process in which acetyl-CoA serves as the donor, transferring an acetyl group to lysine (Lys) or other amino acid residues on target proteins. It is catalyzed by acetyltransferases and regulates gene transcription (26).

#### 2.2.1 Epigenetic regulatory mechanisms of histone acetylation

The dynamic balance between histone acetylation and deacetylation is coordinately maintained by histone acetyltransferases (HATs) and histone deacetylases (HDACs) (27). HATs transfer the acetyl group from acetyl-CoA to lysine residues at the N-terminus of histones, inducing a relaxed chromatin structure that facilitates gene transcription, whereas HDACs catalyze the reverse reaction to repress gene expression (28). HATs can be divided into three major families: GCN5, p300, and MYST. Based on structure and function, HDACs are classified into four classes: Class I (HDAC-1/2/3/8) are nuclear enzymes widely expressed in various tissues; Class II (HDAC-4/5/6/7/9/10) and Class IV (HDAC-11) are mainly localized in the cytoplasm with tissue−specific expression; Class III is the sirtuin (Sir2-related enzymes) family, which are NAD^+^−dependent deacetylases (29). In adult kidney tissue, expression of all Class I and Class II HDAC members can be detected (30). Under the pathological condition of DKD, the promoter regions of fibrosis-related genes exhibit significant enrichment of active chromatin marks along with a reduction in repressive marks. This is accompanied by enhanced binding of HATs and lysine methyltransferases to these regions, collectively driving aberrant transcriptional activation of pro-fibrotic genes (31). Furthermore, abnormal expression or activity of HDACs also participates in the pathogenesis of DKD. HDAC5 is significantly upregulated in glomerular and tubular cells of DM mice and patients, and its knockdown ameliorates HG-induced epithelial-mesenchymal transition (EMT) of TECs (32). TGF-β1 stimulation induces increased nuclear HDAC2 protein expression in TECs and in the kidneys of db/db mice, promoting renal interstitial fibrosis by reducing H3K9 acetylation at the miR-205 promoter (33).

#### 2.2.2 Functional regulation of non-histone protein acetylation

In addition to histones, acetylation of various non-histone proteins indirectly influences gene expression by modulating protein stability, activity, or subcellular localization (34). In renal cells, aberrant acetylation of key metabolic enzymes, transcription factors, and signaling proteins plays an important role in the development of DKD. For example, Rheb1 deficiency promotes mitochondrial dysfunction and accelerates podocyte senescence through enhancing acetylation of the ATP synthase F1 subunit beta (35). Acetylation of the transcription factor NF-κB enhances its transcriptional activity, promotes the expression of inflammatory mediators, and exacerbates renal inflammation (36). Inhibiting deacetylation of transcription factor EB (TFEB) in proximal TECs promotes its nuclear translocation and activation, thereby alleviating tubular injury in DKD by regulating the expression of autophagy-related genes (37).

### 2.3 Ubiquitination

Ubiquitination, a key PTM that regulates protein function, involves the covalent attachment of ubiquitin molecules to target proteins, precisely controlling protein stability, activity, subcellular localization, and molecular interactions, thereby influencing core biological processes such as cell signaling, gene expression, and metabolic remodeling. Ubiquitin is a highly conserved protein composed of 76 amino acids. Its seven internal lysine residues (K6, K11, K27, K29, K33, K48, and K63) can form different types of polyubiquitin chains, mediating diverse signaling pathways (38).

#### 2.3.1 Aberrant degradation by the ubiquitin-proteasome system

The ubiquitin-proteasome system (UPS) controls protein homeostasis by mediating K48-linked polyubiquitination, flagging proteins for 26S proteasome degradation (39). UPS dysfunction in DKD causes abnormal degradation of key regulatory proteins. For example, the E3 ligase RBBP6 mediates K48-linked polyubiquitination of ERRα, promoting its degradation and triggering mitochondrial dysfunction and energy metabolism disorders in TECs (40). The E3 ligase Smurf1 targets NOX4 for K48-linked degradation; thus, inhibiting Smurf1 stabilizes NOX4, increases ROS, and exacerbates renal fibrosis in DKD mice (41). Smurf1 also mediates UPS-dependent degradation of the TGR5 receptor in GMCs under HG conditions, promoting DKD progression (42).

#### 2.3.2 Dysregulation of non-proteolytic ubiquitination signaling

Beyond protein degradation, ubiquitination also regulates protein activity and molecular interactions (43, 44). For example, the deubiquitinase OTUD5 specifically removes K63-linked ubiquitin chains from Lys158 of transforming growth factor-β-activated kinase 1 (TAK1) via its active site C224, thereby inhibiting TAK1-NF-κB signaling activation and alleviating podocyte inflammatory injury (45). Ubiquitin-specific protease 25 suppresses NF-κB and MAPK pathway activation by blocking tumor necrosis factor receptor-associated factor 6-mediated ubiquitination cascades, thereby ameliorating the renal inflammatory microenvironment in DKD (46).

### 2.4 Glycosylation

Glycosylation is the enzymatic or non-enzymatic covalent attachment of carbohydrates to macromolecules. In DKD, non-enzymatic AGEs formation and dynamic O-GlcNAcylation present a dual threat.

#### 2.4.1 Non-enzymatic formation and toxic effects of AGEs

The hyperglycemic DM environment drives non-enzymatic formation of irreversible AGEs (47). AGEs cause renal damage via two mechanisms: direct cross-linking of ECM proteins, leading to glomerular basement membrane thickening/stiffening, and binding to Receptor for Advanced Glycation End Products (RAGE), which activates downstream NF-κB and MAPK signaling, inducing oxidative stress and inflammation (48). Proximal TECs are the primary organ for clearing AGEs, but in DKD, clearance efficiency is diminished by downregulation of AGE receptors. This promotes AGEs deposition in the mesangial area and tubulointerstitium, inducing abnormal ECM proliferation (49). Circulating AGEs levels correlate positively with eGFR decline and all-cause mortality risk in DKD patients; serum AGEs biomarkers can serve as predictive indicators (50).

#### 2.4.2 Dynamic regulation of O-linked N-acetylglucosamine modification

Protein glycosylation in eukaryotes is mainly divided into N-glycosylation and O-glycosylation. Among these, O-linked N-acetylglucosamine modification (O-GlcNAcylation) is a unique form of O-glycosylation (51), dynamically regulated by O-linked N-acetylglucosamine transferase (OGT) (52, 53). Under HG conditions, hyperactivation of the hexosamine biosynthetic pathway leads to accumulation of the donor substrate uridine diphosphate N-acetylglucosamine (UDP-GlcNAc), thereby enhancing OGT-mediated modification. Proteomic studies have shown that HG stimulation significantly elevates O-GlcNAcylation levels of transcription factors and signaling proteins, driving cell proliferation and ECM synthesis (54). O-GlcNAcylation can inhibit phosphorylation signaling by competing for the same Ser/Thr sites, suppressing phosphorylation-dependent activation and contributing to abnormal glomerular hemodynamics (55). Furthermore, enhanced O-GlcNAcylation of heat shock protein 72 impairs its chaperone function, exacerbating protein misfolding stress (56).

### 2.5 Methylation

As core epigenetic mechanisms, DNA methylation and histone methylation regulate DKD onset/progression by reshaping gene expression, deeply involving them in renal fibrosis and metabolic dysregulation.

#### 2.5.1 Aberrant DNA methylation

DNA methylation primarily occurs at CpG islands in gene promoter regions and is catalyzed by DNA methyltransferases (DNMTs) (57). During the pathological progression of DKD, the DNA methylation profile exhibits characteristic alterations. In DKD cohorts from Finland, Denmark, China, and Thailand, leukocytes showed significant hypomethylation in the promoter regions of mechanistic target of rapamycin (mTOR) gene, accompanied by upregulation of gene expression. This suggests that enhanced mTOR complex 1 signaling may contribute to renal hypertrophy (58). Upregulation of TGF-β1 induces hypermethylation of the promoter of RAS protein activator like 1, promoting the development and progression of renal fibrosis (59). The collagen type I alpha 2 chain gene (COL1A2), a key pro-fibrotic gene, exhibits hypomethylation in its gene body region. This hypomethylation is significantly correlated with COL1A2 overexpression in podocytes and proximal TECs of DKD patients, driving excessive ECM deposition (60).

#### 2.5.2 Bidirectional regulation of histone methylation modification

Histone methylation occurs on different lysine residues. The specific residue modified and the degree of methylation determine whether gene expression is activated or repressed. Histone methylation shows dynamic regulation. The active marker H3K4me2 and the repressive marker H3K27me3 can serve as molecular indicators for assessing disease progression (11). In renal tissue of DKD patients, expression of the histone−lysine N−methyltransferase enzyme (EZH2) is upregulated. EZH2 catalyzes the formation of the repressive mark H3K27me3, leading to transcriptional silencing of anti−fibrotic genes (61). Concurrently, the activation mark H3K4me3 is enriched at the promoters of pro−fibrotic genes. This enrichment enhances binding of RNA polymerase II and forms an “epigenetic activation complex”, further aggravating the progression of renal fibrosis (62). These findings reveal the bidirectional role of histone methylation in regulating gene expression in DKD.

### 2.6 SUMOylation

SUMOylation, an important ubiquitin-like PTM, involves an E1-E2-E3 cascade attaching a SUMO protein to target Lys residues. This dynamic process regulates transcription factor activity and protein stability.

#### 2.6.1 Bidirectional regulation of transcription factors by SUMOylation

SUMOylation exerts bidirectional effects on transcription factors in oxidative stress and fibrosis. SUMOylation of Nrf2 inhibits its binding to the antioxidant response element, impairing podocyte oxidative defense and exacerbating oxidative injury (63). Conversely, under hypoxia, SUMOylation of HIF-1α prevents its interaction with the VHL ubiquitin ligase, significantly increasing HIF-1α stability, which promotes transcription of hypoxia-induced genes and accelerates renal fibrosis (64).

#### 2.6.2 Dysregulated SUMOylation of key podocyte proteins and functional impairment

Podocytes, critical for glomerular filtration barrier integrity, are regulated by SUMOylation. In DKD mouse models, abnormally high phosphatase SHP-1 expression reduces SUMOylation of the slit diaphragm protein Podocin, leading to podocyte foot process disorganization, functional impairment, proteinuria, and DKD progression. Genetic SHP-1 deletion restored Podocin SUMOylation and partially reversed DKD pathological changes, underscoring SUMOylation’s importance in maintaining podocyte function (65, 66) and highlighting the complexity of targeting SUMOylation therapeutically.

**Figure 2:**
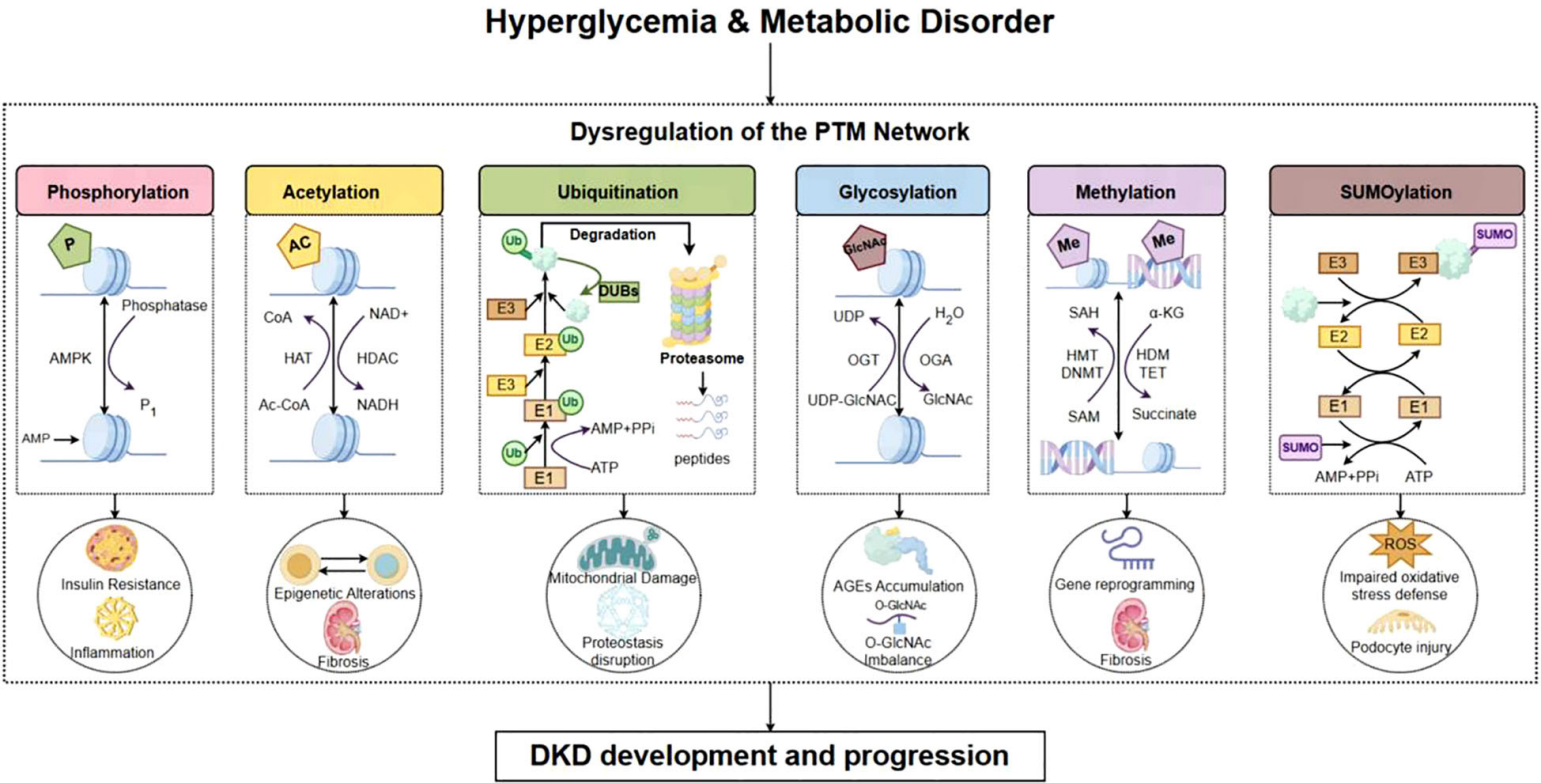
Mechanistic dysregulation and pathological consequences of major PTMs in DKD. Hyperglycemia and metabolic disorders cause systemic dysregulation of six major PTMs. Phosphorylation mediates signaling imbalance and insulin resistance. Acetylation depends on HAT/HDAC equilibrium and contributes to epigenetic disorders and fibrosis. Ubiquitination controls protein stability, and its dysregulation induces mitochondrial damage. Glycosylation is regulated by OGT/OGA and leads to AGEs accumulation and O-GlcNAc disturbance. Methylation mediates gene transcriptional reprogramming. SUMOylation participates in oxidative stress and podocyte injury. Collectively, abnormal PTMs accelerate DKD progression. Created with Figdraw.

Collectively, the molecular mechanisms of the six key PTMs (phosphorylation, acetylation, ubiquitination, glycosylation, methylation, SUMOylation) in DKD share a conserved ‘writer-eraser’ regulatory pattern, as comprehensively illustrated in **Figure 2**. Each modification is orchestrated by specific enzymatic systems that dynamically control its ‘on-off’ state, and their dysregulation converges on multiple pathological processes to drive DKD progression (**Table 1**).

**Table 1:** Summary of PTM regulatory network functions.

PTM type	Key modifying enzymes	Core regulatory mechanism	Pathological effects in DKD
Phosphorylation	Protein kinases (PKC, AMPK, JNK)/Protein phosphatases	Reversible phosphorylation of Ser/Thr residues dynamically regulates protein activity, and modulates core signaling pathways (insulin, MAPK, TGF-β/Smad) (12, 15, 23)	Induces insulin resistance, renal inflammation, ECM deposition, and glomerulosclerosis (14, 18)
Acetylation	Histone acetyltransferases (HATs: p300, CBP)/Histone deacetylases (HDACs: HDAC1-3, SIRT1)	Histone acetylation relaxes chromatin and promotes gene transcription; non-histone acetylation regulates protein stability and signaling activity (26, 27, 34)	Activates pro-fibrotic gene transcription, accelerates cellular senescence, and exacerbates renal inflammation (32, 36)
Ubiquitination	E1/E2/E3 ligases/Deubiquitinating enzymes (DUBs: USP22, USP36)	K48-linked polyubiquitination mediates proteasomal protein degradation; K63-linked chains regulate protein activity and signal transduction (38, 39)	Causes aberrant protein degradation, mitochondrial dysfunction, renal inflammation, and interstitial fibrosis (40, 45)
Glycosylation	OGT/OGA; Non-enzymatic glycation	OOGA dynamically regulates protein function; non-enzymatic formation of AGEs activates RAGE signaling (47, 51, 54)	Induces abnormal glomerular hemodynamics, protein misfolding, oxidative stress, inflammation, and fibrosis (48, 55)
Methylation	DNMTs/EZH2/Demethylases	DNA methylation typically silences gene expression; histone methylation dynamically regulates transcriptional activity (57, 58, 61)	Silences anti-fibrotic genes, activates pro-fibrotic gene transcription, and drives progressive renal fibrosis (59, 62)
SUMOylation	E1/E2/E3 SUMO ligases/SUMO proteases	Modulates transcription factor activity, protein stability, and subcellular localization (63, 64)	Impairs antioxidant defense, accelerates renal fibrosis, and causes podocyte structural and functional damage (65, 66)
Succinylation	Lysine acetyltransferases (KAT2A, p300)/Desuccinylase (SIRT5)	Modifies mitochondrial metabolic enzymes and histones, regulating energy metabolism reprogramming and inflammatory gene expression (67, 68)	Induces mitochondrial lipotoxicity, amplified inflammatory response, and renal lipid accumulation
Lactylation	Histone acetyltransferases (p300)/Delactylase (HDAC1-3)	Driven by glycolytic byproduct lactate, modifies histone H3K18 to activate NF-κB-dependent pro-inflammatory gene transcription (69)	Promotes renal inflammation, tubular EMT, and interstitial fibrosis
Malonylation	Lysine acetyltransferases (p300)/Demalonylase (SIRT5)	Regulates fatty acid metabolic enzyme activity, modulating mitochondrial fatty acid β-oxidation (70, 71)	Exacerbates mitochondrial lipotoxicity, renal lipid accumulation, and oxidative stress
Glutathionylation	Glutathione S-transferases/Glutaredoxins	Redox-sensitive cysteine modification, reversibly regulates protein activity and antioxidant defense (72)	Plays a dual role in renal oxidative stress and inflammatory response
S-nitrosylation	Nitric oxide synthases/Denitrosylases	Nitric oxide-mediated cysteine modification, regulates cell adhesion and mitochondrial function (73)	Disrupts cell-matrix adhesion, mitochondrial dysfunction, and promotes glomerulosclerosis
Palmitoylation	DHHC family palmitoyltransferases/APT1	Transfers palmitoyl groups to cysteine residues, regulating protein membrane localization and signaling activity (74, 75)	Drives renal interstitial fibrosis and tubular EMT
Butyrylation	Histone acetyltransferases (p300, CBP)/Deacetylases (HDAC1-3, SIRT1)	Uses butyryl-CoA as substrate, modifies histone lysine residues to regulate chromatin structure and gene transcription (76)	Inhibits inflammatory gene expression, ameliorates renal fibrosis, and exerts renoprotective effects
β-hydroxybutyrylation (Kbhb)	Histone acetyltransferases (p300, CBP)/Deacetylases (SIRT1-3, HDACs)	Derived from ketone body β-hydroxybutyrate, modifies lysine residues to regulate protein activity and gene transcription (77, 78)	Regulates renal oxidative stress, NLRP3 inflammasome activation, and podocyte injury
Crotonylation	Histone acetyltransferases (p300)/Decrotonylase (SIRT1)	Uses crotonyl-CoA as substrate, modifies histone lysine residues to regulate chromatin remodeling and metabolic reprogramming (79, 80)	Alleviates renal inflammation and fibrosis, inhibits heterochromatin formation at the CD36 promoter

### 2.7 Emerging PTMs

**Figure 3:**
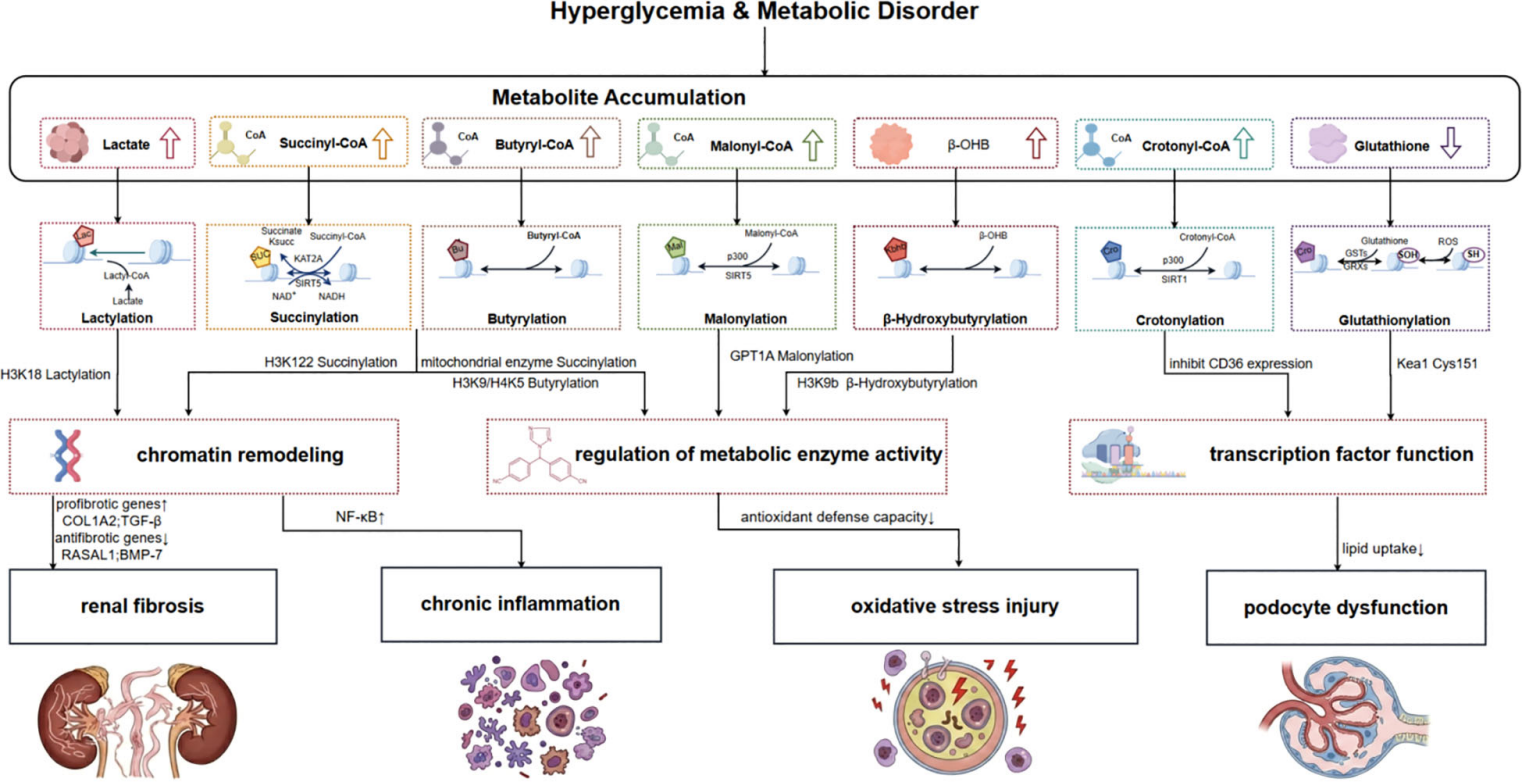
Metabolic-epigenetic coupling and pathogenic signaling driven by emerging PTMs in DKD. Hyperglycemia and metabolic disorders lead to the accumulation of lactate, succinyl-CoA, malonyl-CoA, crotonyl-CoA, butyryl-CoA, and β-hydroxybutyrate. These metabolites mediate a series of emerging PTMs, including lactylation, succinylation, malonylation, crotonylation, glutathionylation, butyrylation, and β-hydroxybutyrylation. By regulating chromatin remodeling, metabolic enzyme activity, transcription factor function, and redox signaling, these PTMs jointly promote fibrotic gene activation, inflammatory responses, oxidative stress, and podocyte dysfunction, thereby aggravating renal injury in DKD. Created with Figdraw.

In DKD progression, beyond classical PTMs, various emerging modifications participate in disease processes by regulating the metabolism-signaling-epigenetics axis (**Figure 3**). Succinylation, an emerging metabolism−related PTM, modifies mitochondrial metabolic enzymes (67) and histones (68), thereby affecting energy metabolic reprogramming and inflammatory gene expression. Lactylation, driven by the glycolytic byproduct lactate, activates NF−κB−dependent pro−inflammatory gene expression through histone H3K18 lactylation (69). Malonylation (70), a key regulator of fatty acid metabolism, is significantly reduced in glycolytic enzymes aldolase A/B in the renal cortex of db/db mice. This reduction is accompanied by decreased malonylation of the fatty acid oxidase SLC27A2, suggesting that malonylation promotes aberrant fatty acid β−oxidation by relieving inhibition of carnitine palmitoyltransferase, thereby exacerbating mitochondrial lipotoxicity (71). S−glutathionylation, a redox−sensitive cysteine (Cys) modification, plays a dual role in DKD oxidative stress. S−glutathionylation of NF−κB p50 Cys62 inhibits its nuclear translocation and alleviates inflammation, whereas glutathionylation of NOX4 Cys495 enhances its ROS−generating capacity and promotes oxidative stress (72). S−nitrosylation, a nitric oxide−mediated cysteine modification, occurs in advanced DKD. VEGF−α induces abnormal nitrosylation of β3−integrin Cys841 and of DLD Cys38, disrupting cell−matrix adhesion and mitochondrial function, thereby promoting glomerulosclerosis (73). Palmitoylation is catalyzed by Aspartic Acid-Histidine-Histidine-Cysteine (DHHC) family palmitoyltransferases, which transfer palmitoyl groups to cysteine residues. DHHC9 and the acyl−protein thioesterase Acyl-protein thioesterases (APT1) drive renal fibrosis by modulating β−catenin palmitoylation (74). The protein acyltransferase DHHC18 promotes renal fibrosis by regulating H−Ras palmitoylation (75).

Beyond the modifications discussed above, recent studies have identified several other short−chain acylations involved in DKD. Butyrylation, a histone modification using butyryl−CoA as a substrate, mediates the renoprotective effects of sodium butyrate in DKD (76). β−Hydroxybutyrylation (Kbhb), derived from the ketone body β−hydroxybutyrate, is elevated under ketogenic or diabetic conditions and regulates oxidative stress and NLRP3 inflammasome activation (77). Notably, hirudin ameliorates DKD by decreasing SOD2 Kbhb levels, thereby reducing ROS and inflammasome formation (78). Crotonylation, another short−chain lysine acylation, exerts a protective effect against renal injury by inhibiting heterochromatin formation at the CD36 promoter, thereby reducing expression of the fatty acid uptake receptor CD36 (79). Sodium crotonate alleviates renal inflammation and fibrosis via the histone crotonylation pathway, representing a potential therapeutic target in DKD (80).

**Figure 4:**
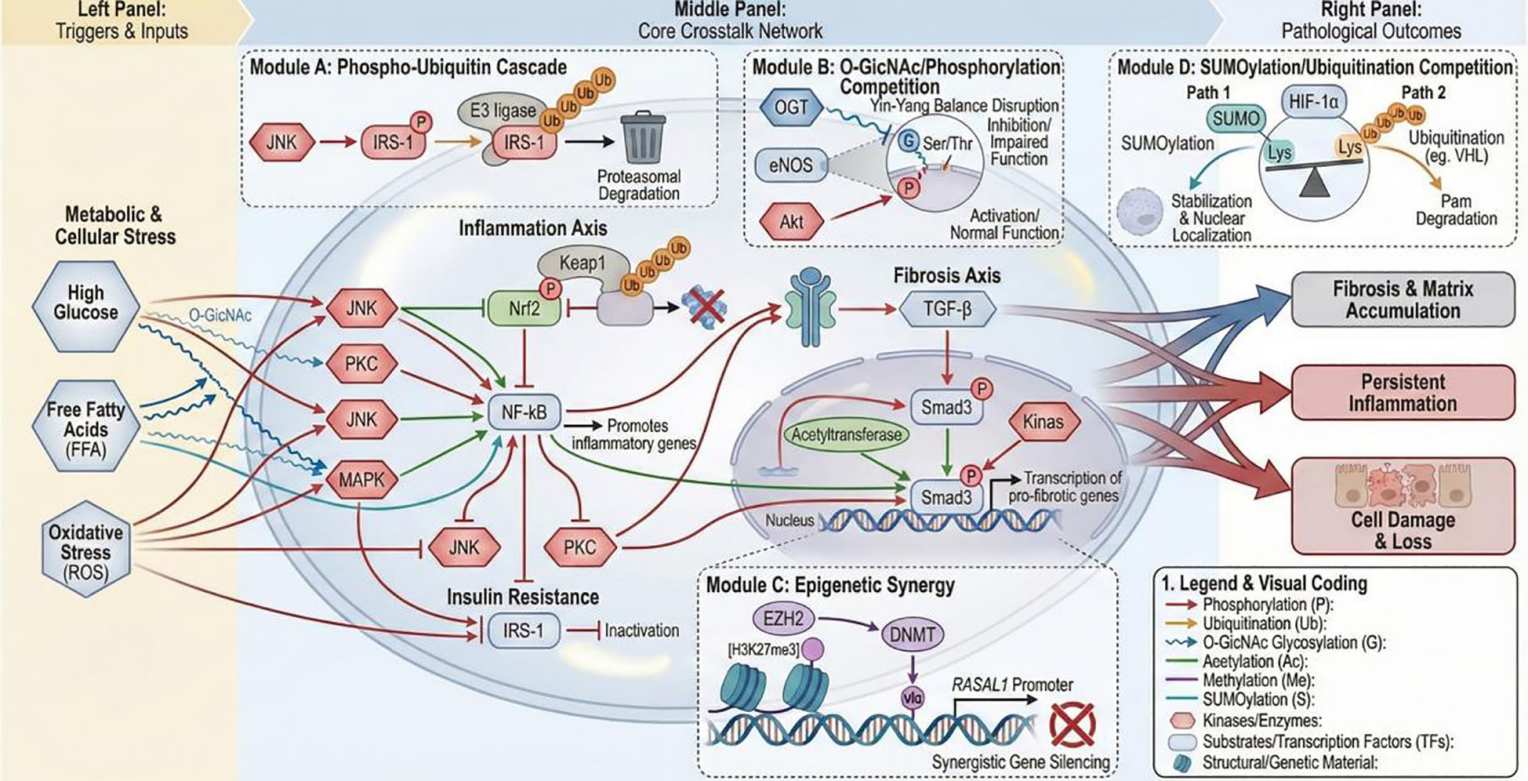
Multidimensional crosstalk network of PTMs in DKD progression. This diagram illustrates the complex crosstalk among various PTMs that links metabolic stress to renal injury. Hyperglycemia, free fatty acids, and oxidative stress activate multiple PTM crosstalk modules: Phosphorylation–ubiquitination crosstalk promotes IRS−1 degradation and insulin resistance; O−GlcNAc/phosphorylation competition disrupts Akt/eNOS signaling; epigenetic synergy between EZH2−mediated methylation and DNMT−mediated methylation silences the RASAL1 gene; SUMOylation/ubiquitination competition modulates HIF−1α stability. These pathways converge on NF−κB−driven inflammation and TGF−β/Smad3−driven fibrosis, ultimately leading to matrix accumulation, inflammation, and renal cell damage in DKD. Created with Figdraw.

These newly identified PTMs constitute a sophisticated regulatory network, and their dysregulation is closely associated with metabolic memory, chronic inflammation, and renal fibrosis in DKD. Integrating multi-omics strategies to dissect dynamic PTM crosstalk will help identify novel biomarkers and therapeutic targets for clinical application. The crosstalk among these PTMs—both classical and emerging—forms an integrated regulatory network that amplifies renal injury, as summarized in **Figure 4**.

## 3 Therapeutic strategies targeting PTMs

Given the central role of PTMs, developing drugs targeting PTM-modifying enzymes is a key frontier. Research focuses on reshaping the aberrant PTM network by modulating enzyme activity, including kinase modulators, HDAC inhibitors, and EZH2 inhibitors, offering a new therapeutic paradigm for DKD intervention (73).

### 3.1 Targeting phosphorylation

Kinases, core enzymes in phosphorylation, are critical nodes driving DKD’s metabolic dysregulation and fibrosis. Developing kinase modulators is a major therapeutic direction. Inhibiting overactive pathways: Since TGF-β/Smad hyperactivation drives fibrosis, Smad3 kinase inhibitors reduce ECM synthesis (23). PKC inhibitors (e.g., ruboxistaurin) show potential to reduce glomerulosclerosis and proteinuria. mTOR inhibitors ameliorate DKD-associated metabolic abnormalities (81). Compounds from traditional herbs (Astragalus mongholicus, Salvia miltiorrhiza, Rehmannia glutinosa) reportedly downregulate PI3K/AKT phosphorylation, restoring podocyte autophagy, inhibiting apoptosis, and exerting renoprotection (82). Sodium-Glucose Cotransporter 2 inhibitors (SGLT2i) exert core renoprotective and cardiovascular protective effects by activating AMPK signaling, which involves the remodeling of multiple PTMs Empagliflozin directly activates AMPK via Thr172 phosphorylation, improving mitochondrial fission, alleviating oxidative stress, enhancing autophagy, and inhibiting fibrotic signaling. As highlighted in the literature (83), AMPK serves as a convergent cardiorenal protective node for SGLT2i, orchestrating crosstalk among phosphorylation, acetylation, O-GlcNAcylation, and other PTMs to simultaneously suppress renal inflammation, fibrosis, and cardiovascular injury. In addition to SGLT2 inhibitors, glucagon-like peptide-1 receptor agonists (GLP-1 RAs) have emerged as another class of glucose-lowering agents with pleiotropic nephroprotective effects. Emerging evidence indicates that GLP-1 RAs exert renoprotection partially through AMPK activation, similar to SGLT2i. Beyond metabolic regulation, GLP-1 RAs attenuate inflammatory damage in DKD by modulating RAGE signaling. Specifically, liraglutide has been shown to remodel the networks of nutrient synthesis and transport, and to promote redox-sensing signals in proximal tubular cells, podocytes, and macrophages, thereby alleviating oxidative stress and inflammation (84). Furthermore, in the proximal tubule, activation of GLP-1 receptor triggers the cAMP signaling cascade, leading to phosphorylation of the Na^+^/H^+^ exchanger isoform 3 (NHE3) and reducing its function. This results in modulated sodium reabsorption and helps maintain blood pressure within the normal range (85). Collectively, these findings position GLP-1 RAs as promising multitarget agents that integrate AMPK-dependent metabolic signaling, RAGE-mediated anti-inflammatory pathways, and cAMP/NHE3-dependent sodium homeostasis to confer nephroprotection in DKD.

### 3.2 Targeting acetylation

HDACs, key enzymes for histone/non-histone deacetylation, are critical targets for correcting epigenetic dysregulation in DKD. Several HDAC inhibitors show renoprotective effects. SIRT1 activation: Resveratrol, a SIRT1 (Class III HDAC) activator, promotes Smad4 deacetylation, inhibiting TGF-β-induced EMT in TECs and mitigating renal fibrosis (86). Resveratrol also improves diabetic energy metabolism by reducing free fatty acid-induced lipotoxicity (87). Sodium butyrate, a broad-spectrum HDAC inhibitor, inhibits HDAC1 and HDAC3, ameliorating renal fibrosis, reducing TECs apoptosis, and lessening oxidative DNA damage (88). Valproic acid inhibits the C5aR-mediated NF-κB pathway, reducing SASP factor release and delaying renal cellular senescence in DKD patients (89). Sulforaphane upregulates H3K27 acetylation at the BMP-7 promoter, reversing its TGF-β-induced epigenetic silencing and improving diabetic renal fibrosis (90). Off-target effects of broad-spectrum HDAC inhibitors are a major obstacle. A new generation of selective HDAC inhibitors is entering clinical trials.

### 3.3 Targeting ubiquitination

The ubiquitination pathway is another key network for therapeutic targeting. Drug discovery targets the entire cascade. E1/E2 Enzymes: Small-molecule activators of E1 restore impaired UPS function in diabetes, alleviating TECs endoplasmic reticulum stress (91). Allosteric E2 inhibitors block K48-linked chain formation and reduce HG-induced inflammatory cytokine release from TECs (92). E3 Ligases/DUBs: E3 ligases are promising targets; e.g., the E3 ligase targeting PPARα for K48-linked degradation drives podocyte injury/proteinuria (93). DUB-modulating drugs are also emerging. Quercetin, a USP22 inhibitor, prevents Snail1 deubiquitination, accelerating its degradation and suppressing TEC EMT (94). USP36 deubiquitinates PLK4 to activate the Wnt/β-catenin pathway, driving abnormal TECs proliferation (95). The Proteasome: The reversible inhibitor MG132 blocks 26S proteasome activity, inducing HSP70 expression and enhancing renal antioxidant capacity, showing potential to prevent DKD development (96, 97). Neddylation Pathway: This ubiquitin-like modification is also a target. NEDD8 is upregulated in diabetic mouse kidneys and HG-stimulated TECs. The selective NEDD8-activating enzyme inhibitor MLN4924 suppresses neddylation, promoting RhoA ubiquitination/degradation and inhibiting renal fibrosis (98, 99).

### 3.4 Targeting glycosylation

Strategies target inhibiting AGEs formation, blocking AGEs-RAGE signaling, and modulating O-GlcNAcylation. AGEs Inhibition: Aminoguanidine, an early AGEs inhibitor trapping reactive dicarbonyl intermediates (100), failed clinical trials due to low bioavailability and toxicity (101). The next-generation inhibitor ALT-946 shows greater potential, improving albuminuria and reducing renal AGEs (102). ACEi (captopril, enalapril) inhibit AGEs-induced PKC activation and alleviate tubular hypertrophy, partly via enhanced NO synthesis (103). RAGE Blockade: Blocking AGEs-RAGE binding (inhibitors: azeliragon, FPS-ZM1) is another strategy (104). Non-coding RNAs also suppress fibrosis/inflammation by regulating RAGE signaling (105). Traditional Chinese medicine ingredients (puerarin, stilbeneglycoside) show multi-target effects by modulating glucose metabolism, antioxidation, inhibiting AGEs, and blocking RAGE (106). O-GlcNAcylation Modulation: Aberrant O-GlcNAcylation elevation is linked to DKD progression. OGT inhibitors reduce HG-induced TGF-β1/fibronectin expression, mitigating fibrosis (107). Conversely, the O-GlcNAcase (OGA) inhibitor Thiamet-G protects podocytes by increasing total O-GlcNAcylation, improving function and reducing proteinuria (108, 109). Notably, SGLT2i (dapagliflozin) indirectly downregulate pathologically elevated O-GlcNAc levels by reducing precursor UDP-GlcNAc availability, a potential novel renoprotective mechanism (110).

### 3.5 Targeting methylation

Overactivation of DNMTs and EZH2 drives DKD by epigenetically silencing anti-fibroticand antioxidant genes. Preclinical studies show therapeutic potential. Low-dose DNMT inhibitor 5-Azacytidine reverses abnormal methylation patterns via genome-wide demethylation, ameliorating HG-induced EMT and renal fibrosis (111). The selective DNMT inhibitor SGI-1027 restores antioxidant/anti-senescence factor expression, improving the renal aging phenotype (112). The EZH2 inhibitor GSK126 protects tubular function by mitigating HG-induced ferroptosis in TECs *in vitro* (61). The EZH2 inhibitor 3-DZNeP reduced oxidative stress/inflammation in TECs in a hyperoxaluria model (113, 114). Precision strategies targeting methylation may offer new hope.

### 3.6 Targeting SUMOylation

SUMOylation’s dual regulatory role makes intervention challenging. The protease SENP6 improves glomerular endothelial cell dysfunction by deSUMOylating/inhibiting Notch1 signaling (115). Conversely, inhibiting the SUMO-activating enzyme subunit 1 reduces HIF-1α stabilization, blocking its downstream pro-fibrotic effects (64). Natural products (astragaloside IV, puerarin, ginkgolic acid) show potential by inhibiting inflammation and mitigating fibrosis, partly by promoting Nrf2 deSUMOylation to enhance its antioxidant activity (116). Significant challenges remain, including poor tissue selectivity and incomplete understanding.

### 3.7 Targeting emerging PTMs

Therapeutic strategies targeting regulatory enzymes for emerging PTMs are emerging. These PTMs are involved via a metabolism−epigenetics−oxidative stress axis. In the context of metabolism, lactylation links glycolysis and epigenetics. IGFBP5 promotes EMT and fibrosis by upregulating H3K18 lactylation (117), whereas GLIS1 inhibits TECs senescence and fibrosis by downregulating histone lactylation (118). Modulating histone lactylation may therefore represent a novel therapeutic strategy. Regarding oxidative stress, S−glutathionylation and malonylation reversibly regulate protein activity. Glutathione activates Nrf2 and induces substrate S−glutathionylation, mitigating HG−induced β−cell dysfunction (72). The demalonylase SIRT5 corrects abnormal fatty acid oxidation by reducing malonylation across non−mitochondrial pathways (71). In terms of lipidation, chlorogenic acid alleviates renal fibrosis by reducing lipid accumulation via Notch1/STAT3 inhibition (80). Sodium crotonate, which induces H3K18 crotonylation, suppresses inflammatory gene expression and lowers blood glucose and lipid levels in DKD mouse models (119). These strategies are still in early exploration, and their safety and efficacy require extensive investigation.

### 3.8 Multi-target combinations

**Figure 5:**
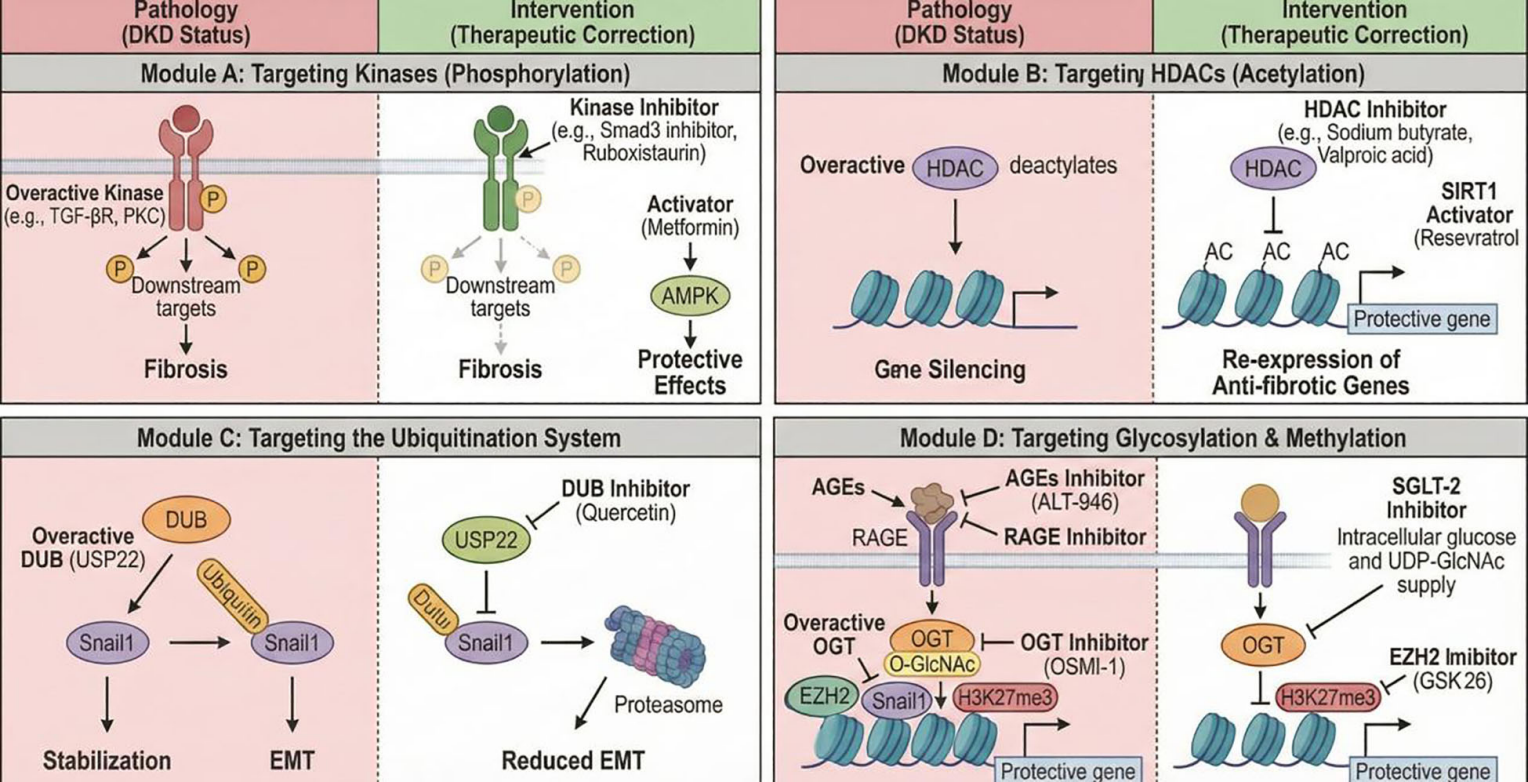
Mechanistic targets for precision intervention: Rebalancing dysregulated PTM networks in DKD. This figure illustrates four key therapeutic modules for correcting dysregulated PTM networks in DKD. Targeting kinase signaling: inhibiting overactive kinases (e.g., TGF-βR, PKC) or activating AMPK signaling ameliorates fibrosis. Targeting acetylation: HDAC inhibitors or SIRT1 activators restore histone acetylation and reactivate anti-fibrotic genes. Targeting ubiquitination: inhibiting USP22 promotes Snail1 degradation and suppresses EMT. Targeting glycosylation and methylation: blocking AGEs, OGT, or EZH2, combined with SGLT-2 inhibitors, normalizes metabolic-epigenetic signaling. These interventions collectively rebalance PTM networks to alleviate renal injury. Created with Figdraw.

Given the PTM network’s complexity, redundancy, and compensatory regulation, single-target interventions may have limited efficacy or face resistance. Multi-target or combination strategies are a critical direction, aiming for synergistic effects by intervening at multiple nodes. Combining PTM Modulators: Combining an HDAC inhibitor (sodium butyrate) with a PI3K/Akt activator. The HDAC inhibitor increases histone acetylation, while the PI3K/Akt activator improves IR/podocyte function. They can synergistically reduce inflammation, decrease proteinuria, and delay fibrosis (120). The microbial metabolite indole-3-propionic acid exerts renoprotection by concurrently regulating three PTMs: activating SIRT1 (promoting deacetylation), inhibiting GSK-3β Ser9 phosphorylation, and reducing β-catenin ubiquitination/degradation, comprehensively alleviating DKD (121, 122). Combining PTM-Targeted and Conventional Drugs: Metformin with an AGEs inhibitor achieves better glycemic control, reduces AGEs formation, and modulates metabolism/oxidative stress, enhancing renoprotection (123). The modular therapeutic strategies targeting PTM dysregulation, as comprehensively visualized in **Figure 5**, demonstrate the feasibility of precision intervention in DKD (**Table 2**).

**Table 2:** Classification of targeted therapy drugs.

Target PTM	Drug/compound	Direct molecular target	Mechanism of action & Renoprotective effect	Stage
Phosphorylation	Ruboxistaurin (81)	PKC	Inhibits PKC; reduces glomerulosclerosis and proteinuria.	Phase 2 Clinical Trial
	Metformin (123)	AMPK	Activates AMPK; alleviates metabolic dysregulation and enhances autophagy.	Clinically Approved
	Empagliflozin (83)	SGLT-2	Inhibits SGLT-2, activating AMPK; reduces mitochondrial fission.	Clinically Approved
Acetylation	Resveratrol (86)	SIRT1	Activates SIRT1; inhibits EMT in tubular cells.	Preclinical/Phase 1 Clinical Trial
	Sodium Butyrate (88)	HDAC1/3	Inhibits HDACs; restores histone acetylation and ameliorates fibrosis.	Preclinical
	Valproic Acid (89)	HDACs	Inhibits HDACs; delays cellular senescence.	Clinically Approved for Other Indications
Ubiquitination	MLN4924 (98)	NEDD8-Activating Enzyme	Inhibits neddylation pathway; suppresses renal fibrosis.	Preclinical
	Quercetin (94)	USP22	Inhibits DUB activity; suppresses EMT in tubular cells.	Preclinical
	MG132 (97)	26S Proteasome	Inhibits proteasome; enhances renal antioxidant capacity.	Preclinical (Tool compound)
Glycosylation	ALT-946 (102)	AGEs Formation	Inhibits dicarbonyl intermediates; reduces AGEs accumulation.	Preclinical
	Captopril (103)	ACE	Inhibits ACE; suppresses AGE-induced PKC activation.	Clinically Approved
	OSMI-1 (107)	OGT	Inhibits OGT; mitigates renal fibrosis.	Preclinical
	Thiamet-G (109)	OGA	Inhibits OGA; increases protective O-GlcNAcylation in podocytes.	Preclinical
Methylation	5-Azacytidine (111)	DNMTs	Inhibits DNMTs; reverses aberrant DNA methylation patterns.	Preclinical
	SGI-1027 (112)	DNMTs	Inhibits DNMTs; ameliorates renal senescence phenotypes.	
	GSK126 (61)	EZH2	Inhibits EZH2; protects tubular cells from ferroptosis.	
SUMOylation	Astragaloside IV (116)	SUMOylation enzymes	Modulates SUMOylation (e.g., Nrf2 deSUMOylation); enhances antioxidant defense.	Preclinical
Butyrylation	Sodium Butyrate (120)	HDAC1/3	Inhibits HDAC-mediated debutyrylation, increases histone butyrylation, suppresses inflammatory gene expression, and ameliorates renal fibrosis	Preclinical
β-hydroxybutyrylation	β-hydroxybutyrate (77)	Histone Acetyltransferases	Increases histone β-hydroxybutyrylation, regulates oxidative stress and NLRP3 inflammasome activation, and protects podocytes	Preclinical
Crotonylation	Sodium Crotonate (80)	Histone Crotonylation	Induces histone crotonylation, suppresses inflammatory gene expression, lowers blood glucose/lipid levels, and alleviates renal fibrosis	Preclinical

## 4 Clinical translation challenges and future perspectives

Despite the tremendous promise of PTM-targeted therapies, significant clinical bottlenecks in DKD remain unresolved. These include the relentless progression to ESRD even with optimal glucose and blood pressure control, a persistently high residual risk of cardiovascular events, the lack of early and specific diagnostic biomarkers, and the inability to reverse established renal fibrosis. Current PTM-based strategies have not yet overcome these obstacles, primarily due to systemic off-target toxicity, the complexity of the PTM network with its redundant and compensatory mechanisms, and the absence of non-invasive tools for real-time PTM monitoring (124). Therefore, highlighting these shortcomings is not a weakness of the field but rather a necessary step to underscore the urgent need for deeper mechanistic insights and innovative translational approaches. This review, by systematically mapping the regulatory networks of PTMs in DKD, provides a foundational framework to address these gaps. In parallel, emerging clinical applications of PTMs, such as circulating O−GlcNAcylated proteins as early biomarkers for DKD progression and histone acetylation patterns as predictive indicators for drug response, are beginning to show promise. Fundamental research, powered by multi−omics technologies (e.g., PTM proteomics, single−cell mass cytometry) (125) and advanced genetic models, is now dissecting the dynamic PTM “modificome” and revealing previously unrecognized therapeutic nodes (126, 127). Collectively, bridging the gap between fundamental discoveries and clinical needs will be essential to transform PTM−targeted strategies from experimental concepts into real−world solutions for DKD patients (**Table 3**). In summary, by systematically dissecting the regulatory networks of PTMs and honestly appraising their current limitations in overcoming clinical bottlenecks of DKD, this review serves as both a roadmap for fundamental research and a catalyst for clinical translation.

**Table 3:** Clinical translation challenges and strategies of PTM-targeted therapy in DKD.

Clinical translation bottleneck	Specific manifestation in DKD	Potential resolution strategy
Off-target toxicity (124)	Broad-spectrum HDAC inhibitors cause thrombocytopenia; p38 MAPK inhibitors increase infection risk	Kidney-specific delivery (nanocarriers, cell-penetrating peptides)
Complexity of combination therapy (125)	Unpredictable PK/PD interactions; risk of excessive metabolic suppression	Systems biology and AI-driven modeling; adaptive trial designs
Lack of sensitive efficacy endpoints (126, 127)	Traditional endpoints (eGFR, proteinuria) change slowly; no real-time PTM monitoring	Molecular biomarkers (e.g., urinary O-GlcNAcylated proteins); mass cytometry (CyTOF); near-infrared fluorescence imaging probes

## 5 Conclusion

DKD is a progressive disease driven by complex metabolic, epigenetic, and signaling disorders. Post-translational modifications serve as critical molecular switches that connect metabolic stress to renal injury. This review systematically summarizes the regulatory networks of classical and emerging PTMs in DKD, highlights their roles in inflammation, fibrosis, and mitochondrial dysfunction, and discusses prospects for clinical translation. These insights provide a theoretical basis for developing novel diagnostic markers and targeted therapies for DKD.

## Glossary

Ac: Acetylation
Ac-CoA: Acetyl-Coenzyme A
AGEs: Advanced Glycation End Products
AMP: Adenosine Monophosphate
AMPK: AMP-Activated Protein Kinase
α-KG: α-Ketoglutarate
ATP: Adenosine Triphosphate
BMP-7: Bone Morphogenetic Protein 7
CD36: Cluster of Differentiation 36
CoA: Coenzyme A
COL1A2: Collagen Type I Alpha 2 Chain
DKD: Diabetic Kidney Disease
DNMT: DNA Methyltransferase
DM: diabetes mellitus
DUB: Deubiquitinating Enzyme
eNOS: Endothelial Nitric Oxide Synthase
ECM: Extracellular matrix
EMT: Epithelial-Mesenchymal Transition
ESRD: end-stage renal disease
EZH2: Enhancer of Zeste Homolog 2
Glc: Glycosylation
GlcNAc: N-Acetylglucosamine
GMC: Glomerular Mesangial Cell
GPT1A: Glycerol-3-Phosphate Acyltransferase 1, Mitochondrial
GRXs: Glutaredoxins
GSTs: Glutathione S-Transferases
HAT: Histone Acetyltransferase
HDAC: Histone Deacetylase
HDM: Histone Demethylase
HG: High glucose
HIF-1α: Hypoxia-Inducible Factor 1-Alpha
HMT: Histone Methyltransferase
IRS-1: Insulin Receptor Substrate 1
JNK: c-Jun N-Terminal Kinase
KAT2A: Lysine Acetyltransferase 2A
Keap1: Kelch-Like ECH-Associated Protein 1
MAPK: Mitogen-Activated Protein Kinase
Me: Methylation
NAD+: Nicotinamide Adenine Dinucleotide
NF-κB: Nuclear Factor Kappa B
Nrf2: Nuclear Factor Erythroid 2-Related Factor 2
OGA: O-GlcNAcase
OGT: O-GlcNAc Transferase
P: Phosphorylation
PKC: Protein Kinase C
PPi: Pyrophosphate
PTM: Post-Translational Modification
RAGE: Receptor for AGEs
ROS: Reactive Oxygen Species
SAH: S-Adenosylhomocysteine
SAM: S-Adenosylmethionine
SGLT-2: Sodium-Glucose Cotransporter-2
SIRT: Sirtuin
SIRT1: Sirtuin 1
Smad3: SMAD Family Member 3
SUMO: Small Ubiquitin-Like Modifier
TECs: Tubular Epithelial Cells
TET: Ten-Eleven Translocation Methylcytosine Dioxygenase
TGF-β: Transforming Growth Factor-Beta
TGF-βR: Transforming Growth Factor-Beta Receptor
Tyr: Tyrosine
Ub: Ubiquitin/Ubiquitination
UDP: Uridine Diphosphate
UDP-GlcNAc: Uridine Diphosphate N-Acetylglucosamine
USP22: Ubiquitin-Specific Peptidase 22
VHL: von Hippel-Lindau Tumor Suppressor.
